# Data article on the effectiveness of entrepreneurship curriculum contents on entrepreneurial interest and knowledge of Nigerian university students

**DOI:** 10.1016/j.dib.2018.03.011

**Published:** 2018-03-08

**Authors:** Maxwell Olokundun, Oluwole Iyiola, Stephen Ibidunni, Mercy Ogbari, Hezekiah Falola, Odunayo Salau, Fred Peter, Taiye Borishade

**Affiliations:** Covenant University, Nigeria

**Keywords:** Entrepreneurship curriculum contents, Entrepreneurial interest and knowledge, University students, Nigeria

## Abstract

The article presented data on the effectiveness of entrepreneurship curriculum contents on university students’ entrepreneurial interest and knowledge. The study focused on the perceptions of Nigerian university students. Emphasis was laid on the first four universities in Nigeria to offer a degree programme in entrepreneurship. The study adopted quantitative approach with a descriptive research design to establish trends related to the objective of the study. Survey was be used as quantitative research method. The population of this study included all students in the selected universities. Data was analyzed with the use of Statistical Package for Social Sciences (SPSS). Mean score was used as statistical tool of analysis. The field data set is made widely accessible to enable critical or a more comprehensive investigation.

**Specification Table**

**Table:** 

**Subject area**	Business, Management
**More Specific Subject Area:**	Business and Entrepreneurship education
**Type of Data**	Table
**How Data was Acquired**	Researcher-made questionnaire analysis
**Data format**	Raw, analyzed, Inferential statistical data
**Experimental Factors**	Sample consisted of university students in Nigeria. The researcher-made questionnaire which contained data on entrepreneurship curriculum contents, students’ entrepreneurial interest and knowledge were completed.
**Experimental features**	Entrepreneurship curriculum contents are one of the factors endangering entrepreneurial development of university students.
**Data source location**	South west Nigeria
**Data Accessibility**	Data is included in this article

**Value of data**

● These data present descriptive data on entrepreneurship curriculum contents in universities as it relates to development of entrepreneurial interest and knowledge by undergraduates. This is geared towards development of salient entrepreneurial competencies by university students.
● The results showed that the use of practical oriented curriculum contents can be very helpful for universities in designing curriculum contents for entrepreneurship education.
● The results of this study can be used to improve teaching and learning practices in university entrepreneurship education.

## 1 Data

As shown in Table 1 below a total of six hundred copies of questionnaire were administered to the students of four selected pioneer universities to offer a degree in entrepreneurship in Nigeria. Five hundred and sixty-four copies of the questionnaire were retrieved, which amounted to a 94% response rate. Five hundred and sixty-four copies of the questionnaire retrieved were found useable and a total of thirty six copies of the questionnaire were not retrievable, which amounted to 6%.

**Table 1:** Response rate of copies of questionnaire administered.

Questionnaire	Number of respondents	Response rate (%)
Returned	564	94
Not Returned	36	6
Total	600	100

Based on the copies of questionnaire retrieved, below is the demographic information showing the distribution based on age gender and educational qualification on Table 2.

**Table 2:** Distribution of biographical data of the respondents.

Demographic Variables		University 1		University 2		University 3		University 4		Total	Percentage
		Freq	%	Freq	%	Freq	%	Freq	%		%
Gender:	M	29	46.0	55	38.5	93	48.7	107	64.1	284	50.4
	F	34	54.0	88	61.5	98	51.3	60	35.9	280	49.6
Age:	15–19	19	30.2	48	33.6	100	52.4	94	56.3	261	46.5
	20–24	33	52.4	84	58.7	84	44.0	69	41.3	270	47.9
	Above 25years	11	17.5	11	7.7	7	3.7	4	2.4	33	5.6
Degree Programme	B.sc/B.A	36	57.1	121	84.6	177	92.7	63	37.7	397	70.4
	B.Tech/Eng	5	7.9	14	9.8	11	5.8	99	59.3	129	22.9
	B.Ed/Others	22	34.9	8	5.6	3	1.6	5	3.0	38	6.7

### 1.1 Gender distribution

Table 2 above shows the frequency distribution of respondents’ demographic data. The distribution of gender reveals that male respondents were 284(50.4%) and female respondents were 280 (49.6%). Despite the 0.8% difference between the two genders, data obtained represents a rich and balanced opinion of both genders. University 4 had the highest number of male respondents (107) representing 37.7% of the total number of male respondents and University 1 had the lowest number of male respondents (29) representing 10.2% of the total number of male respondents. On the other hand, University 2 had the highest number of female respondents (98) representing 35% of the total number of female respondents and University 1 had the lowest number of female respondents (34) representing 12.1% of the total number of female respondents. This validates the even distribution of respondents based on gender.

### 1.2 Age distribution

The age distribution revealed that 261 (46.5%) were respondents between ages 15 to 19 years, 270 (47.9%) were respondents between ages 20 to 24 years, and 33 (5.6%) were respondents above 25 years. The result indicates that most of the respondents were between the ages 20–24 years (270) representing 47.9% of the total number of respondents. However, both University 2 and 3 shared the same top number 84 each of the respondents between the ages 20–24 representing 31.1% each respectively. Respondents within the age bracket above 25 years were the minority, with University 4 having the lowest number of respondents in this age bracket (4) representing 0.7% of the total number of respondents. This implies that most respondents offering entrepreneurship education within the university context are mostly between the ages 20–24 years. This also shows that most of the respondents are young adults who can independently give informed responses.

### 1.3 Degree programme

Information provided by respondents in Table 2 on degree programme of respondents shows that 397 (70.4%) were B.Sc/B.A students, 129 (22.9%) were B.Tech/Eng students, and 38 (6.7%) were B.Ed/Other students. The degree programme results revealed that more of the respondents were B.Sc./B.A students (397) followed by B.Tech/Eng students 129 and the least were B.Ed/Other students 38. However, the distribution of degree programme of respondents cuts across different disciplines, which implies that the opinions of respondents from different disciplines were considered.

Table 3 above reveals that when respondents were asked if better understanding about business is achieved as a result of taking this course, most of the respondents answered positively to the statement. The analysis in the table shows that the mean scores of the respondents from University 1, 2, 3, and 4, are 4.3532, 4.3636, 4.0733, and 3.8443 respectively. On the other hand, respondents from university 1 and 2 agreed more favourably to the statement with mean scores 4.3532 and 4.3636 respectively. This suggests that more respondents from university 1 and 2 opine that their understanding of business and entrepreneurship has been broadened as a result of participation in entrepreneurship education. The table shows that when respondents were asked if the course developed entrepreneurial knowledge and skills, most respondents favourably agreed to the statement. The analysis shows that the mean scores of the respondents from university 1, 2, 3, and 4 are 4.2576, 4.3776, 4.0105, and 4.0240 respectively. This implies that more students from university 2 and 1 with mean scores 4.3776 and 4.2576 respectively, believe that the entrepreneurship course has inculcated entrepreneurial skills and knowledge in students that they did not possess prior to exposure to the course. The table also reveals that most respondents affirmed that the course raised interest towards entrepreneurship. The analysis revealed that the mean scores for university 1, 2, 3, and 4 are 4.3117, 4.3287, 3.9529, and 3.9461, respectively.

**Table 3:** Descriptive statistics of items measuring entrepreneurship curriculum contents based on each selected university.

**Statement**	**University 1**	**University 2**	**University 3**	**University 4**
	**Mean score**	**Mean score**	**Mean score**	**Mean score**
Better understanding about business is achieved as a result of taking the course	4.3532	4.3636	4.0733	3.8443
The course developed entrepreneurial knowledge and skills	4.2576	4.3776	4.0105	4.0240
The course raised interest towards entrepreneurship	4.3117	4.3287	3.9529	3.9461

This result suggests that more respondents from university 1 and 2 with mean scores 4.3117 and 4.3287 respectively, are of the opinion that students have developed interest in engaging in entrepreneurial activities based on the information and knowledge acquired from the entrepreneurship course. Findings from the descriptive statistics showed that most respondents agreed that the entrepreneurship course enhanced better understanding about business and it developed entrepreneurial knowledge and skill [2]. The descriptive statistics also revealed that most respondents agreed that the course developed entrepreneurial knowledge and skills [13]. Furthermore, the findings from the descriptive statistics revealed that most respondents agreed that the course raised interest towards entrepreneurship. These results are in consonance with the findings of [5] who suggested that the design of an entrepreneurship curriculum may stimulate the development of entrepreneurial knowledge and the practice of entrepreneurship. This is in line with the work of [6] and [14] who asserted that the provision of university curricular content on idea generation has implications for the development of entrepreneurial interest and skills of learners. It is also confirms the findings of [11] which showed that the design of an entrepreneurship curriculum affects entrepreneurial learning outcomes. Similar to many other studies in the field of management, the findings of this research is limited on the basis of generalization since a few universities with unique characteristics were sampled for the study.

### 1.4 Study area description

Nigeria is the world's eighth leading exporter of oil, and Africa's second largest economy, following South Africa [1]. Nigeria also represents 15 per cent of Africa's population, and contributes 11 per cent of Africa's total output as well as 16 per cent of its foreign reserves, accounting for half of the population and more than two-thirds of the total output of West Africa sub-region (World Population Prospects [15]). Nigeria is the most heavily populated nation in Africa, which is naturally blessed with millions of acres of arable land, thirty eight billion barrels of state oil reserves, large gas reserves, an assortment of unused and untapped mineral resources, and a wealth of manpower and human capital by reason of its estimated population of above 160 million people [4].

Entrepreneurship education programmes in Nigerian Universities was the focus of this study. Specifically, the study examined the effects of entrepreneurship curriculum contents on students’ development of entrepreneurial interest and knowledge. However, emphasis was laid on the first four universities in Nigeria to offer a Bachelors degree programme in entrepreneurship. The entrepreneurship programmes in these universities were considered relevant to the context of this study because there are indications that best practices in entrepreneurship education are obtainable in these universities, and also because the main aim of the entrepreneurship programmes in these institutions is to motivate students to initiate entrepreneurial actions during the course of the programmes. Attention was given the perceptions of students in the selected universities. This provided a basis to understand how students interpret the teaching and learning processes in entrepreneurship education and how these affects their behavioural responses and actions.

## 2 Experimental design, materials and methods

Four universities were selected from South west Nigeria. Six hundred students in the selected universities participated in this study. Data were gathered from students across the various colleges in the selected universities with the aid of a researcher- made questionnaire based on the works of [3], [7], [8], [9], [10], and [12]. The demographic data presented information based on gender.age and degree programme as well as questions related entrepreneurship curriculum contents and the development of entrepreneurial interest and knowledge. There was a meaningful relationship between entrepreneurship curriculum contents and the development of entrepreneurial interest and knowledge in the selected universities in south west Nigeria. The collected data were coded and entered into SPSS version 22. Data analysis was performed; using SPSS-22.Data was analyzed applying descriptive statistical tests which involved mean scores.

Statistical Package for Social Sciences may be inadequate to address some advanced modeling and development of statistical approaches however, for a simple analysis such as descriptive analysis; the key selling point is its expansive data analysis options. What makes it even better is that these functions are automated to the point that one simply needs to select the applicable variables and matching applications for output and analysis and the package does the rest.

## 3 Conclusion and implications of the study

The propensity for university graduates to become job creators may largely depend on the extent to which the design of an entrepreneurship curriculum stimulates students’ entrepreneurial interest and knowledge. This has implications for university managements and other stakeholders as regards the design of entrepreneurship curriculums. Therefore, the data described in this article is made widely accessible to facilitate critical or extended analysis.
